# Heavy Chalcogen Properties of Sulfur and Selenium Enhance Nucleic Acid-Based Therapeutics

**DOI:** 10.3390/biom15020218

**Published:** 2025-02-02

**Authors:** Stephen J. Dansereau, Jia Sheng

**Affiliations:** Department of Chemistry and the RNA Institute, University at Albany, State University of New York, Albany, NY 12222, USA

**Keywords:** chalcogens, nucleic acids, sulfur, selenium

## Abstract

The Group 16 elements of the periodic table have a characteristic valence shell configuration instrumental to their chemical properties and reactivities. The electrostatic potentials of these so-called chalcogens have been exploited in the design of materials that require the efficient passage of electrons including supermagnets, photocatalytic dyes, and solar panels. Likewise, the incorporation of the heavy chalcogen selenium into organic frameworks has been shown to increase the reactivities of double bonds and heterocyclic rings, while its interactions with aromatic side chains in the hydrophobic core of proteins via selenomethionine impart a stabilizing effect. Typically present in the active site, the hypervalence of selenocysteine enables it to further stabilize the folded protein and mediate electron transfer. Selenium’s native occurrence in bacterial tRNA maintains base pair fidelity, most notably during oxidative stress, through its electronic and steric effects. Such native modifications at the positions 2 and 5 of uridine render these sites relevant in the design of RNA-based therapeutics. Innocuous selenium substitution for oxygen in the former and the standard methods of selenium-derivatized oligonucleotide synthesis and detection have led to the establishment of a novel class of therapeutics. In this review, we summarize some progress in this area.

## 1. Chemical Properties of Chalcogens as Sources of Innovation

**Figure 1:**
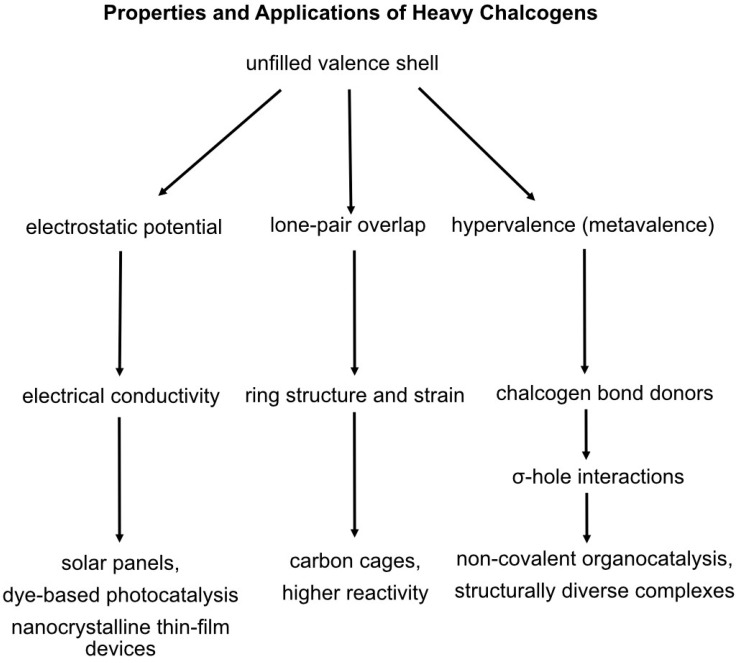
Flow diagram of chalcogen properties and applications. Unfilled valence shells of heavy chalcogens impart these elements with unique properties conducive to various applications.

Chalcogens belong to Group 16 of the periodic table and include oxygen, sulfur, selenium, tellurium, and polonium. Common among these elements is their need for two additional electrons to satisfy their electronic shell closure. However, the coulomb repulsion of added electrons prevents heavier chalcogens below oxygen in Group 16 from retaining the second electron, thus imparting these elements with unique properties [1]. A flowchart of these atomic characteristics and related applications is presented in Figure 1.

## 2. Chalcogen Electrostatics Improve Material Design

One property of chalcogens is their magnitudes of negative electrostatic potentials, resulting in greater polarities and consequent reactivities than highly electronegative oxygen [2]. These heavy chalcogens have been exploited in the rational design of super-atoms, i.e., clusters of homo- or hetero-atoms, to enhance the properties of materials and even customize nanoscale assemblies [3]. Advances have been made in mitigating the formation of oxychalcogenide mixtures and oxygen-containing byproducts [4] to create lattices displaying superconductivity and the fractional quantum Hall effect [5].

The same electrostatic potentials have been exploited in organic frameworks to enhance the electrical conductivity of light harvested from the sun [6] and facilitate the passage of electrons across stacked aromatic rings in dye-based photocatalysis [7]. Fluorescence dyes, in particular, are capable of phototuning by interchanging chalcogen atoms, and such one-atom differences allow the resulting probes to conform to physiological sites of interest [8].

Likewise, the superior interparticle charge transfer dynamics of surface-passivating phenyl chalcogenol ligands from cadmium selenide quantum dots have led to an improved performance of nanocrystalline thin-film devices [9]. Thiophene, dithiophene, and thienothiophene diboronic acids have already been reported to engage in unique charge transfer interactions with electron acceptors. Substituting sulfur with selenium or tellurium in exciton diffusion materials should amplify charge mobility due to their 3p- or 4p-orbital overlap, respectively, or their inflated spin–orbit coupling effects [6].

## 3. Metallo-Heterocyclic Rings Exhibit Greater Stability

This overlap among lone pairs with the pi orbitals of triple bonds also enables heavy chalcogens to modulate the properties of hydrocarbon rings. Further, given the energy difference between orbitals, selenium and polonium are known to decrease ring strain, even in three-membered acetylene units [10]. For instance, the ring architecture of metallacyclopropabenzenes contains much less strain than their non-metallic counterparts owing to the longer and softer metal–carbon bonds than carbon–carbon bonds. In fact, a three-membered ring containing a heavy metal such as selenium or tellurium is more readily able to anneal with benzene to form a stable product [11].

Another means of influencing ring structure is in the form of carbon cages, in which heavy chalcogens are incorporated at the epicenter of various membered rings [12]. As endohedral atoms, they are capable of storing energy to later enthalpically drive forward reactions [13]. Encapsulated fullerene rings, in particular, are the speculated origins of novel molecules and materials [14].

1,2-dichalcogen heterocycles have many pharmaceutical uses including chemotherapy and antioxidant and radiation protection, and they can be used as chemopreventive, choleretic, and sialagogue agents. Wu et al. (2020) used a [3+2] cycloaddition to insert elemental selenium into the heterocycle in a similar reaction that incorporated elemental sulfurs into 1,2-diathiole-3-thiones, which is a template for the known therapeutics oltipraz, S-Danshensu, and NOSH-1 [15].

## 4. Heavy Chalcogen Incorporation Primes Organic Reactivity

Chalcogens’ high nucleophilicities also enable them to be easily incorporated into synthetic compounds [16]. Once assimilated into molecules, consistent with their role in carbon cages, chalcogens’ reactivities are in turn enhanced [17]. Similarly, the incorporation of heavy chalcogens to generate heteroallenes enhances the reactivity of double bonds [18].

Further contributing to this group’s reactivity is their hypervalence, sometimes termed metavalence to emphasize their deviation from ordinary covalent, ionic, and metallic bonding [19], which has been used to investigate the bonding nature of tetravalent chalcogens [20]. Heavy chalcogens also serve as donor atoms to dihalogens and interhalogens to form complexes with broad structural diversity [21].

Chalcogen bond donors facilitate non-covalent organocatalysis though σ-hole interactions, in which a half-filled p-orbital creates a positive electrostatic potential [22], thereby decreasing the HOMO-LUMO gap of the reactants [23]. The term chalcogen bond has been coined to describe donors such as Lewis acids [24,25], thus in a fashion analogous to hydrogen bonds [26,27].

Selenium is capable of forming hydrogen bonds when incorporated into the molecular framework of therapeutics. For instance, the selenium atom of an analog of anti-hypertensive captopril was shown to hydrogen bond with angiotensen-1-converting enzyme at the backbone of histidine-367 [28].

## 5. Chalcogen-Derivatized Therapeutics and Dietary Selenium Are Anticancerous

Replacing oxygen, and even sulfur, with heavier chalcogens provides a means of tuning the photocatalysis of porphyrin-based drugs, experimentally improving their efficacy against certain cancers [29]. Such a substitution into photosensitizers increases their excitation wavelengths to a tissue-appropriate range, enabling their transcutaneous induction at the tumor site [30]. Selenium- and tellurium-containing genopyrylium dyes have demonstrated in vitro phototoxicity against mammary adenocarcinoma cells and the inhibition of mitochondrial cytochrome c oxidase [31].

The anticancer properties of organoselenium compounds ingested from cruciferous vegetables suggests that synthetic isoselenocyanates could be administered therapeutically [32]. Moreover, polysulfide [33] and organoselenium substitutions [34] within hydroxytyrosol 2-(3,4-dihydroxyphenyl)ethanol, the component of extra virgin olive oil linked to lower risks of cardiovascular disease and certain cancers [35], further improve this polyphenolic’s antioxidant and anticancer capabilities [36].

Selenium-enriched polysaccharide extracts from Ganoderma mushrooms proved to be superior free radical scavengers [37], and these trace minerals [38] enter the food chain through the uptake of selenate and selenite by plants and the subsequent conversion to organic forms [39,40].

## 6. Geologic Source and Biologic Nature of Chalcogens in Proteins and Nucleic Acids

The chalcogens sulfur, selenium, and tellurium have been sampled from nebular condensates as components of chondrules [41], though delivery to Earth by meteors is unlikely the sole source of them given the low atmospheric estimates of these elements [42]. The establishment of volatile chalcogens in the crust following protoplanetary differentiation [43] and the separation of liquid sulfide from sulfur-saturated magmas provide alternate sources of chalcogens that have fractionated within the mantle [44]. The subsequent uptake of these chalcogens by plants in the forms of sulfate and selenate and the trophic transfer of their organic forms [45] imbue higher organisms with their unique biochemical properties.

## 7. Sulfated Amino Acids Stabilize Molecular Interactions

The lower electronegativity of sulfur than oxygen makes for unstable hydrogen bonding, rendering the side chain of methionine hydrophobic in nature [46]. One caveat of this hydrophobicity is the versatility with which the sulfur atoms of methionine engage the face of the planar aromatic rings of certain side chains [47], thereby stabilizing the hydrophobic core and also making an enthalpic contribution to this folding process [48].

Rosenfield’s (1977) re-examination of published crystal structures touted the divalent sulfur of methionine as pivotal in engaging both aromatic residues in a fixed orientation and polar oxygen atoms [49]. In fact, these weak, nonbonded sulfur–oxygen interactions are another stabilizing force in the protein structure [50] and can lead to the formation of a chalcogen bond.

Surveys of the Protein Data Bank have highlighted the stabilizing potential of intramolecular chalcogen bonding, which is assumed when the distance between oxygen and sulfur is too short for van der Waals contact [51]. The main chain carbonyl oxygen, side chain carboxyl oxygen, and the histidine side chain nitrogen all partake in nucleophilic attack with the divalent sulfur of methionine [52]. This process’s directional selectivity is influenced by a cooperativity involving peripheral, weak interactions [53], with an overall geometry unique to biopolymers [54].

**Figure 2:**
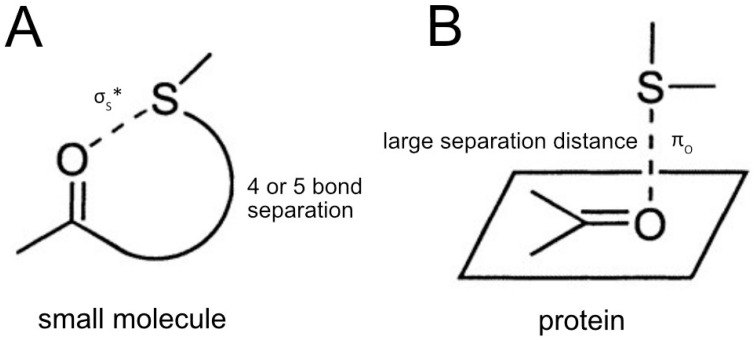
The orientation of the nucleophilic attack of oxygen on sulfur. The relatively close proximity of nucleophilic oxygen to sulfur in organic compounds demands a backside attack known as one oriented in the σ_S_* direction (**A**). By contrast, the larger distance separating these two atoms in proteins allows for a perpendicular orientation of attack termed the π_O_ direction (**B**). Adapted with permission from [57]. Copyright 2002. American Chemical Society.

The typical four- or five-bond separation of sulfur from oxygen in organic compounds demands a backside approach of nucleophilic oxygen toward sulfur in what is termed the σS* direction [55], drawn in Figure 2A. By contrast, the sulfurs of methionine and cysteine approach the main chain carbonyl oxygen perpendicular to the amide plane, illustrated in Figure 2B, in the so-called πO direction, a feature afforded by the larger distance between interacting moieties [55]. The resultant electrostatic surface, characteristic of sulfur-mediated heterocycles, governs the biologic properties of these proteins [56].

Intermolecular chalcogen bonding has been shown to facilitate catalysis [58] and mediate anion transport [59]. The relatively strong chalcogen bond of a sulfonium cation bound to oxygen permits the lysine methyltransferase SET7/9 to recognize its AdoMet substrates [60]. It is believed that the substitution of sulfur with selenium retains these binding interfaces [61], and the native presence of selenium can, in fact, augment biologic activity [62,63].

## 8. Selenoproteins Facilitate Cellular Redox Reactions

Many bacterial enzymes involved in electron transfer [64] contain selenocysteine in their active sites [65], such that selenium serves to coordinate iron–nickel metal centers [66]. The advantages of this are illustrated by [NiFeSe] hydrogenase: the acidity of the selenogroup makes the nickel–hydride bond more labile and protects the active site from inactivation via oxygen [67]. Further, selenium-containing hydrogenases are readily activated under reductive conditions [68], enabling selenoenzymes such as thioredoxin reductase [69], glycine reductase [70], and glutathione peroxidase [71] to scavenge free radicals released from the respiratory chain [72].

Vital to the synthesis of these antioxidative proteins, selenoprotein P can also deleteriously release excess selenium into the cell [73], causing glucose intolerance and insulin resistance [74]. In addition, selenoprotein P is a biomarker [75] in pulmonary hypertension [76] and has been proposed as a multifaceted therapeutic target [77].

Previewing their diverse roles in human endocrinology [78], another selenoprotein containing an FAD prosthetic group was discovered from human lung adenocarcinoma by Tamura et al. (1996) that catalyzes the NADPH-dependent reduction of 5,5′-dithiobis(2-nitrobenzoic acid) and the reduction of insulin [79]. Likewise, a highly reactive selenocysteine is required for the catalysis of type I iodothyronine 5′-deiodinase to produce thyroid hormone in Rana catesbeiana tissues [80], involving a selenenyl iodide intermediate [81].

The nucleophilicity of selenium in the active site supports a 1000-fold greater catalysis than its cysteine replacement [82], and, unlike thiyl radicals, selanyl radicals do not oxidize aromatic amino acids [83]. Further contributing to protein stability is selenium’s ability to hydrogen bond [84]: the hydrogen bond donors cysteine and selenocysteine exert a stabilizing effect through interactions with main chain amino groups [85,86]. Main chain carbonyl groups are also capable of hydrogen bonding with nucleic acids [87], and hydrogen bonding among the latter is attributed to their base pair specificity [88,89].

## 9. Mnm Enzymes Catalyze Sulfur and Selenium Substitution Within Nucleic Acids

Selenium-substituted nucleic acids have comparable base pairing ability to their canonical counterparts [90], a feature influenced by base stacking and solvent interactions [91]. Selenium’s broad-based stabilizing effects on stacking interactions [92], as well as reports of this atom’s large size hampering this stacking interaction [90], leave the former mechanism unresolved. The latter is curbed by selenium’s weak hydrogen bonding ability, while the electronic and steric effects of selenium enhance base pair discrimination [93].

These very properties are used in nature at the 2-exo position of uridine, replacing the 2-exo oxygen with selenium, in order to destabilize the promiscuous U/G wobble pair in favor of the canonical U/A [94]. Such substitutions are common among bacterial tRNAs, namely tRNALys, tRNAGlu, and tRNAGln [95], and selenium’s strong nucleophilicity along with the dipole character of the Se+-O bond [96] allows seleno-tRNAs to maintain translational fidelity under oxidative stress [97].

**Figure 3:**
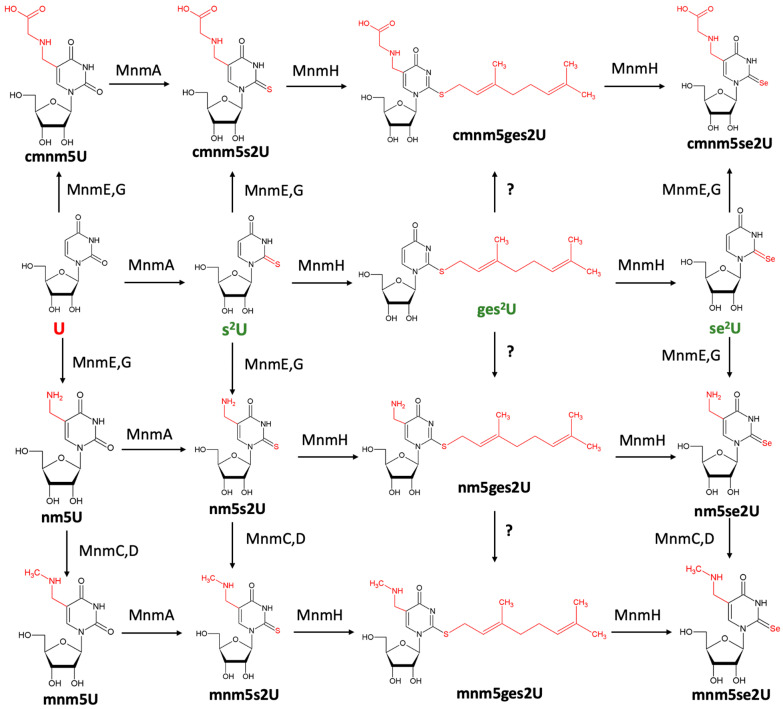
Intertwined methyltransferase biosynthetic pathways. Post-transcriptional, hypermodified tRNA is formed through the activity of several enzymes in order to decrypt degenerate codons for accurate translation. Likewise, chalcogen derivatization at the 2-position provides favorable redox chemistry. MnmH is an alternate name for SelU. Question marks means the enzyme for the reaction is not known.

Lighter sulfur also offers redox capability to tRNA [98], and its substitution at the 2-exo position of uridine, in conjunction with a methylaminomethyl substitution at the 5-position, is an alternate tool nature uses to stabilize the anticodon stem loop [99]. The consequent substitution-specific rigidity at the wobble position enables the accurate translation of degenerate codons, namely, those coding for the amino acids glutamate, glutamine, lysine, arginine, and glycine [57,100]. Such post-translational hypermodifications employ the activity of several enzymes [101], some of which are mapped to their respective biosynthetic pathways in Figure 3.

Intermediates and products along these tRNA hypermodification pathways are present in humans and bacteria along with analogous enzymes. For instance, bacterial MnmA and eukaryotic cytosolic Ncs6 synthesize s2U34, without which translational fidelity would be lost among otherwise thermophilic bacteria, and human frameshift mutations would result in myoclonus epilepsy [102]. Mechanistically, MnmA is a thiouridylase that transfers sulfur from L-cysteine to its own catalytic cysteine and ultimately onto the 2-uridine of tRNA [103].

Further catalysis with MnmE is thought to catalyze one of the steps involved in the synthesis of cmnm5s2U34 [104], with the carboxy methylamino group originating from glycine and the carbon appended to the 5-position borrowed from tetrahydrofolate [105]. The subsequent FAD-dependent oxidative cleavage of the carboxymethyl group followed by an SAM-dependent methylation, both catalyzed by MnmC, yields mnm5s2U34 [106]. The dependence of 5-uridine derivatization on MnmD and MnmG, which act in concert with MnmC and MnmE, respectively, can also be observed in Figure 3.

**Figure 4:**
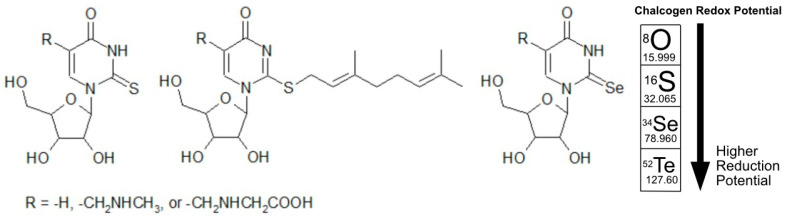
Chalcogen redox properties facilitate translation. The higher reduction potential of selenium enables such derivatized tRNA, most notably at the anticodon wobble position, to retain base pair fidelity in the presence of reactive oxygen species.

MnmH is unique to bacteria and believed to be instrumental in both the 2-uridine S-geranylation and selenation steps [107], each offering unique attributes in base pairing and redox chemistry. The latter can be tailored to the environmental conditions by swapping sulfur for selenium [108], as illustrated in Figure 4. Perhaps a more descriptive name for the MnmH protein tasked with this conversion is tRNA-2-selenouridine synthase (SelU), which through a two-step mechanism replaces 2-sulfur with selenium [109].

The catalytic intermediate contains an S-geranyl group at this 2-position [110], conferring an enhanced recognition of the codons ending in the nucleobase guanine [111]. The further stability of the duplex arises from the insertion of the 2-terpene group into the minor groove [112]. The subsequent selenation at this position diminishes the hydrogen bond network with guanine owing to selenium’s steric interference and 2-selenouridine’s preferential hydrogen bonding with adenine [94].

We recently found this mechanism of catalysis to involve a cooperativity among SelU’s N- and C-terminal domains, containing respective binding sites for 2-thiouridine-tRNA and geranyl pyrophosphate. The binding of the latter is required for the transfer of its geranyl group to 2-thiouridine-tRNA, thereby allowing S-geranyl-modified tRNA to form a stable complex with the N-terminal domain of SelU. Thus, a plausible inhibitor may include a nucleobase or nucleoside scaffold containing moieties from each of these ligands. Further competition for the tRNA-binding site can be achieved by substituting the 2-exo-sulfur of this mimetic’s uridine base with a heavier chalcogen.

## 10. Chalcogens in Nucleic Acid Mimicry and Their Detection via Analytical Methods

Replacing the oxygen of nucleic acids with a heavier chalcogen, be it sulfur, selenium, or even tellurium, offers a means of tuning the aforementioned bonding and redox properties for therapeutic applications [113,114]. A variety of selenium-derivatized nucleic acids have already been shown to be chemically stable and biologically active [115]. For instance, neither synthetic 2′-methylseleno guanosine nor selenium-derivatized cytidine exhibit any effects on RNA packing or A-form DNA duplex formation, respectively [116,117]. Moreover, the native C3′-endo conformation of A-form DNA and RNA molecules is retained following substitution with selenium at the 2′-α-position [118].

Positioning selenium at the 4′-position has been proposed for third-generation nucleosides displaying antiviral and antitumor properties [119], and Lee et al. (2019) replaced the nucleoside furanose ring oxygen with selenium to generate the novel anti-HCV agent 2′-C-methyl-4′-selenopyrimidine [120]. The strategic placement of selenium within such frameworks has been proposed to guard against a number of tropical diseases [121] and leishmaniasis-causing parasites [122].

A geranyl group, shown as part of an MnmH catalytic intermediate in Figure 3, offers a dual moiety that can be exploited to further advance nucleoside-based therapeutics. Derivatization at the nucleoside sugar or base may be sufficient to mimic the 2-S-geranyluridine intermediate while retaining the signature selenium. In fact, assigning this atom to the 2-exo position would offer a novel nucleoside scaffold to serve as a broad-spectrum antibiotic.

## 11. Native Uridine Modifications Customize Small Molecule Mimetics

The 5-position of the uridine nucleobase presents an alternate site of modification owing to the influence of native substitutions at this position over translation and isoform selectivity [123,124]. These heterocycles commonly contain 5-methylcarboxymethyl (mcm5U) and 5-methylaminomethyl (mnm5U) moieties [125,126], which nature has had billions of years to evolve [127]. However, provided the de novo synthesis of functional macromolecules [128,129], the activity of selenated nucleosides can be steered by incorporating native and semi-native moieties at the 2- and 5-nucleobase positions.

**Figure 5:**
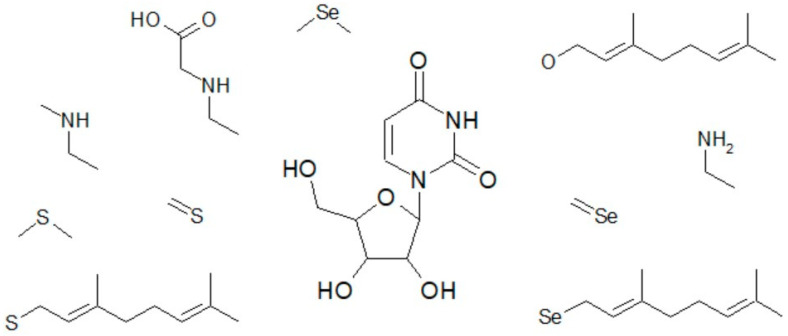
Derivatized uridine as a pharmacophore. Various combinations of native and de novo substituents at the sugar and base can be explored for their pharmacologic potencies.

Starting from the standard nucleoside uridine, a small library of nucleoside-based pharmacophores can be devised through unique combinations of six total native 2- and 5-uridine modifications, illustrated in Figure 3. One set of nucleosides leaves the 5-position singly protonated, while another contains the native 2-carbonyl oxygen. A rationally designed antibiotic may entail repositioning these native moieties among the sugar and base, illustrated in Figure 5, or adding de novo functional groups.

Using SelU as a drug target, this proposed library can be screened for a derivatized uridine with sufficient potency to disrupt bacterial translation at the wobble position. Thus, four ways to design a therapeutic that mimics tRNA at the wobble position in certain bacteria involve placing at the 2-uridine either oxygen, sulfur, S-isoprenoid, or selenium. Though reports also seem to implicate the native geranyl group in base pair discrimination and receptor recognition, a novel analog might exploit the properties of both selenium and terpene at 2-uridine. This de novo 2-selenogeranyl-uridine, unlike the native 2-uridine substituents, contains attributes of both steps of SelU catalysis, theoretically bolstering its affinity to this enzyme.

Likewise, a carboxymethylaminomethyl (cmnm), aminomethyl (nm), methylaminomethyl (mnm), or a single proton substituted at the 5-uridine position provide further variability while imitating the native substituents. The broad-spectrum antiviral activities of 2-thiouridine analogs have already been reported [130], while the efficacy of 6-thioguanine against inflammatory disease and cancer suggests the utility of other nucleobases as pharmacophores [131].

Yielding the desired product is not a trivial task, as it involves the precise positioning of functional groups and reactive chalcogens. The canonical nucleoside template can be synthesized or commercially purchased, to which a selenium atom is substituted at oxygen in order to best mimic the native counterpart [118].

## 12. Selenium-Derivatized Phosphoramidites in Synthetic Oligomers Drive Crystal Growth

Positioning this selenium, whether on the nucleoside sugar [132] or base, requires protecting and activating groups under optimized conditions [133]. Upon deprotection, using selenide or methylselenide is a common means of selenium incorporation into the now primed position [134]. As the side products may consist of an enantiomeric mix, the separation of the desired diastereomer can be achieved via anion-exchange HPLC [135] with the subsequent molecular weights of these derivatized nucleosides obtained via MALDI-TOF mass spectrometry [136].

The conversion of this selenium-derivatized nucleoside to a stable phosphoramidite [137] lays the foundation for the production of selenium-containing DNA and RNA oligonucleotides through solid-phase synthesis [138]. Though a synthetic approach limits the size of a given oligonucleotide strand, enzymatic ligation methods are available to conjoin selenium-enriched strands [139], as in the production of antisense oligonucleotides [116]. The higher melting temperature of these RNA duplexes connotes the stability conferred by the selenium modification [140], which also allows such DNA and RNA to better withstand X-ray irradiation during crystallization [141].

The presence of the heavy atom selenium not only promotes crystal packing and growth across a range of buffer conditions [142] but also serves as a remedy for the phasing problem [143]. Standard diffraction measurements yield an amplitude for each of the thousands of diffracted waves [144], leaving the phase information to be computationally derived or experimentally determined [145].

The phasing method of choice is multi-wavelength anomalous diffraction (MAD) owing to its collection of a data set encompassing the full array of wavelengths, thereby yielding a more accurate phase calculation [146,147]. This approach requires so-called phase atoms exhibiting an X-ray absorption edge within the experimentally defined energy range [148]. Moreover, these phase atoms must be present in both a sufficient quantity and at regularly spaced intervals within the polymer [149].

The selenium K absorption edge is conducive to obtaining readily interpretable electron density maps [150], and unlike halogens, it is amenable to all four RNA nucleosides [151]. Thus, the derivatization of DNA and RNA with selenium is a promising strategy for detecting oligonucleotides and elucidating their structures using X-ray crystallography [152].

## 13. Conclusions

Heavy chalcogens have been investigated for their use in commercial products that bring comfort and convenience, as well as in organic frameworks that drive chemical reactions and exhibit pharmaceutical efficacy. These attributes are mirrored by their native presence as selenomethionine, selenocysteine, and selenouridine in certain macromolecules, by which they serve vital roles in cellular redox and translational fidelity. The latter has been well documented in bacteria, highlighting the enzyme involved in selenating uridine as a plausible drug target.

The catalysis of MnmH, better known as SelU, can serve as a case study for biologically pertinent moieties that can be exploited in the optimization of nucleoside mimetics. Already, third-class nucleosides containing selenium have been developed as antimicrobial agents. Equipping a uridine scaffold at various positions with either the moieties of biosynthetically mapped intermediates or de novo functional groups is a promising strategy in the rational design of such a pharmacophore. Although synthetic modifications to nucleoside sugars have been documented, the conjoining base presents additional sites worth exploring.

Natively substituted positions are ideal for derivatization owing to their physiological relevance, and our laboratory is exploring various combinations of substituents at the 2- and 5-nucleobase positions of uridine. Of particular interest is the incorporation of selenium, as occurs in the final step of the SelU-catalyzed reaction, to create either a small molecule or RNA therapeutic.

The conversion of the former to a phosphoramidite and subsequent oligonucleotide has become standard protocol, as the presence of selenium improves the macromolecule’s stability and detection via X-ray crystallography. Moreover, the transposition of selenium for oxygen abides by native structural and physiological constraints while allowing for the design of therapeutics with enhanced properties.

## References

[1] Hassan A.E., Sheng J., Jiang J., Zhang W., Huang Z. (2009). Synthesis and crystallographic analysis of 5-Se-thymidine DNAs. Org. Lett..

[2] Hassan A.E., Sheng J., Zhang W., Huang Z. (2010). High fidelity of base pairing by 2-selenothymidine in DNA. J. Am. Chem. Soc..

[3] Henriquez-Figuereo A., Moreno E., Sanmartin C., Plano D. (2023). Exploring Novel Drug Combinations: The Therapeutic Potential of Selanyl Derivatives for Leishmania Treatment. Molecules.

[4] Mayo R.A., Morgan I.S., Soldatov D.V., Clerac R., Preuss K.E. (2021). Heisenberg Spin Chains via Chalcogen Bonding: Noncovalent S-O Contracts Enable Long-Range Magnetic Order. Inorg. Chem..

[5] Kim J., Almo S.C. (2013). Structural basis for hypermodification of the wobble uridine in tRNA by bifunctional enzyme MnmC. BMC Struct. Biol..

[6] Bartholomew A.K., Meirzadeh E., Stone I.B., Koay C.S., Nuckolls C., Steigerwald M.L., Roy X. (2022). Superatom regiochemistry dictates the assembly and surface reactivity of a two-dimensional material. J. Am. Chem. Soc..

[7] Henriquez-Figuereo A., Morán-Serradilla C., Angulo-Elizari E., Sanmartín C., Plano D. (2023). Small molecules containing chalcogen elements (S, Se, Te) as new warhead to fight neglected tropical diseases. Eur. J. Med. Chem..

[8] Christofferson A., Zhao L., Sun H., Huang Z., Huang N. (2011). Theoretical studies of the base pair fidelity of selenium-modified DNA. J. Phys. Chem. B.

[9] Berseneva A.A., Klepov V.V., Pal K., Seeley K., Koury D., Schaeperkoetter J., Wright J.T., Kanatzidis M.G., Berseneva A.A., Gelis A.V. (2022). Transuranium Sulfide via the Boron Chalcogen Mixture Method and Reversible Water Uptake in the NaCu T S3 Family. J. Am. Chem. Soc..

[10] Halder A., Data D., Seelam P.P., Bhattacharyya D., Mitra A. (2019). Estimating strengths of individual hydrogen bonds in RNA base pairs: Toward a consensus between different computational approaches. ACS Omega.

[11] Chand A., Sahoo D.K., Rana A., Jena S., Biswal H.S. (2020). The prodigious hydrogen bonds with sulfur and selenium in molecular assemblies, structural biology, and functional materials. Acc. Chem. Res..

[12] Beckett G.J., Arthur J.R. (2005). Selenium and endocrine systems. J. Endocrinol..

[13] Wittwer A.J., Tsai L., Ching W.M., Stadtman T.C. (1984). Identification and synthesis of a naturally occurring selenonucleoside in bacterial tRNAs: 5-[(methylamino) methyl]-2-selenouridine. Biochemistry.

[14] Kitamura A., Sengoku T., Nishimoto M., Yokoyama S., Bessho Y. (2011). Crystal structure of the bifunctional tRNA modification enzyme MnmC from *Escherichia coli*. Protein Sci..

[15] Nawrot B., Sierant M., Szczupak P. (2023). Sulfur-and Selenium-Modified Bacterial tRNAs. Handbook of Chemical and Biology of Nucleic Acids.

[16] Kadlag Y., Becker H. (2016). Highly siderophile and chalcogen element constraints on the origin of components of the Allende and Murchison meteorites. Meteorit. Planet. Sci..

[17] Wang Z., Becker H. (2013). Ratios of S, Se and Te in the silicate Earth require a volatile-rich late veneer. Nature.

[18] Wang Z., Becker H. (2015). Fractionation of highly siderophile and chalcogen elements during magma transport in the mantle: Constraints from pyroxenites of the Balmuccia peridotite massif. Geochim. Cosmochim. Acta.

[19] Benz S., Macchione M., Verolet Q., Mareda J., Sakai N., Matile S. (2016). Anion Transport with Chalcogen Bonds. J. Am. Chem. Soc..

[20] Biswal H.S. (2015). Hydrogen bond involving sulfur: New insights from ab initio calculations and gas phase laser spectroscopy. Noncovalent Forces.

[21] Bock A. (2006). Selenium proteins containing selenocysteine. Encyclopedia of Inorganic Chemistry.

[22] Carlson B.A., Lee B.J., Tsuji P.A., Copeland P.R., Schweizer U., Gladyshev V.N., Hatfield D.L. (2018). Selenocysteine tRNA^[Ser]Sec^, the central component of selenoprotein biosynthesis: Isolation, identification, modification, and sequencing. Selenoproteins: Methods and Protocols.

[23] Carlson B.A., Xu X.M., Kryukov G.V., Rao M., Berry M.J., Gladyshev V.N., Hatfield D.L. (2004). Hatfield. Identification and characterization of phosphoseryl-tRNA^[Ser]Sec^ kinase. Proc. Natl. Acad. Sci. USA.

[24] Pal D., Chakrabarti P. (2001). Non-hydrogen bond interactions involving the methionine sulfur atom. J. Biomol. Struct. Dyn..

[25] Wilds C.J., Pattanayek R., Pan C., Wawrzak Z., Egli M. (2002). Selenium-assisted nucleic acid crystallography: Use of phosphoroselenoates for MAD phasing of a DNA structure. J. Am. Chem. Soc..

[26] Höbartner C., Rieder R., Kreutz C., Puffer B., Lang K., Polonskaia A., Serganov A., Micura R. (2005). Syntheses of RNAs with up to 100 Nucleotides Containing Site-Specific 2 ‘-Methylseleno Labels for Use in X-ray Crystallography. J. Am. Chem. Soc..

[27] Morgado C.A., McNamara J.P., Hillier I.H., Burton N.A., Vincent M.A. (2007). Density functional and semiempirical molecular orbital methods including dispersion corrections for the accurate description of noncovalent interactions involving sulfur-containing molecules. J. Chem. Theory Comput..

[28] Payne N.C., Geissler A., Button A., Sasuclark A.R., Schroll A.L., Ruggles E.L., Gladyshev V.N., Hondal R.J. (2017). Comparison of the redox chemistry of sulfur- and selenium-containing analogs of uracil. Free Radic. Biol. Med..

[29] Bethune D.S., Johnson R.D., De Vries M.S., Yannoni C.S. (1993). Atoms in carbon cages: The structure and properties of endohedral fullerenes. Nature.

[30] Root M.J., Deutsch E. (1981). Nucleophilicity of coordinated chalcogens as evaluated in DMF-water media. Inorg. Chem..

[31] Behne D., Kyriakopoulos A., Meinhold H., Köhrle J. (1990). Identification of type I iodothyronine 5’-deiodinase as a selenoenzyme. Biochem. Biophys. Res. Commun..

[32] Duhovic S., Dinca M. (2015). Synthesis and electrical properties of covalent organic frameworks with heavy chalcogens. Chem. Mater..

[33] Xue D., Wu D., Chen Z., Li Y., Sun W., Liu J., Li Z. (2021). On Close Parallels between the Zintl-Based Superatom Ge9Be and Chalcogen Elements. Inorg. Chem..

[34] Garcin E., Vernede X., Hatchikian E.C., Volbeda A., Frey M., Fontecilla-Camps J.C. (1999). The crystal structure of a reduced [NiFeSe] hydrogenase provides an image of the activated catalytic center. Structure.

[35] Deacon A.M., Ealick S.E. (1999). Selenium-based MAD phasing: Setting the sites on larger structures. Structure.

[36] Ealick S.E. (2000). Advances in multiple wavelength anomalous diffraction crystallography. Curr. Opin. Chem. Biol..

[37] Pallan P.S., Egli M. (2007). Selenium modification of nucleic acids: Preparation of phosphoroselenoate derivatives for crystallographic phasing of nucleic acid structures. Nat. Protoc..

[38] Frieben E.E., Amin S., Sharma A.K. (2019). Development of isoselenocyanate compounds’ syntheses and biological applications. J. Med. Chem..

[39] Bader R.F., Fang D.C. (2005). Properties of atoms in molecules: Caged atoms and the Ehrenfest force. J. Theory Comput..

[40] Ouhsaine F., Ranaivonjatovo H., Escudié J., Saffon N., Lazraq M. (2009). From a Phophagermaallene -P=C=Ge< and Heavier Chalcogens (S, Se, Te): Access to 3-Phosphanylidene-1,2,Chalcogenagermiranes. Organometallics.

[41] Knight F.R., Fuller A.L., Bühl M., Slawin A.M., Woollins J.D. (2010). Hypervalent adducts of chalcogen-containing peri-substituted naphthalenes; reactions of sulfur, selenium, and tellurium with dihalogens. Inorg. Chem..

[42] Visioli F., Galli C. (1998). Olive oil phenols and their potential effects on human health. J. Agric. Food Chem..

[43] Chen G., Zhao T., Wang Q., Jena P. (2019). Rational design of stable dianions and the concept of super-chalcogens. J. Phys. Chem. A.

[44] Rose G.D., Geselowitz A.R., Lesser G.J., Lee R.H., Zehfus M.H. (1985). Hydrophobicity of amino acid residues in globular proteins. Sci. Adv..

[45] Werz D.B., Gleiter R. (2004). [*N*]Chalcogena[*N*]pericycynes: DFT Studies on Binaric Carbon-Chalcogen Compounds. Org. Lett..

[46] Jäger G., Chen P., Björk G.R. (2016). Transfer RNA bound to MnmH protein is enriched with geranylated tRNA-A possible intermediate in its selenation. PLoS ONE.

[47] Waska H., Kim S., Kim G., Kang M.R., Kim G.B. (2008). Distribution patterns of chalcogens (S, Se, Te, and 210Po) in various tissues of a squid, *Todarodes pacificus*. Sci. Total Environ..

[48] Karas C., Hecht M. (2020). A strategy for combinatorial cavity design in de novo proteins. Life.

[49] Wang M.S., Hecht M.H. (2020). A completely de novo ATPase from a combinatorial protein design. J. Am. Chem. Soc..

[50] Hendrickson W.A. (2014). Anomalous diffraction in crystallographic phase evaluation. Q. Rev. Biophys..

[51] Moroder H., Kreutz C., Lang K., Serganov A., Micura R. (2006). Synthesis, oxidation behavior, crystallization and structure of 2’-methylseleno guanosine containing RNAs. J. Am. Chem. Soc..

[52] Maroney M.J., Hondal R.J. (2018). Selenium versus sulfur: Reversibility of chemical reactions and resistance to permanent oxidation in proteins and nucleic acids. Free Radic. Biol. Med..

[53] Caton-Williams J., Huang Z. (2008). Biochemistry of selenium-derivatized naturally occurring and unnatural nucleic acids. Chem. Biodivers..

[54] Sheng J., Huang Z. (2008). Selenium derivatization of nucleic acids for phase and structure determination in nucleic acid X-ray crystallography. Int. J. Mol. Sci..

[55] Sheng J., Huang Z. (2010). Selenium derivatization of nucleic acids for X-ray crystal-structure and function studies. Chem. Biodivers..

[56] Sun H., Sheng J., Hassan A.E., Jiang S., Gan J., Huang Z. (2012). Novel RNA base pair with higher specificity using single selenium atom. Nucleic Acids Res..

[57] Iwaoka M., Takemoto S., Tomoda S. (2002). Statistical and theoretical investigations on the directionality on nonbonded S-O interactions. Implications for molecular design and protein engineering. J. Am. Chem. Soc..

[58] Hunter M.S., Yoon C.H., DeMirci H., Sierra R.G., Dao E.H., Ahmadi R., Aksit F., Aquila A.L., Ciftci H., Guillet S. (2016). Selenium single-wavelength anomalous diffraction de novo phasing using an X-ray-free electron laser. Nat. Commun..

[59] Lee H., Jarhad D.B., Yu J., Lee C., Jeong L.S. (2019). 2’-C-Methyl-4’-selenonucleosides as Anti-Hepatitis C Virus Agents. J. Org. Chem..

[60] Fowler J.E., Schaefer H.F. (1994). The tetramethyl chalcogens (Me4S, Me4Se, Me4Te): Bonding and structure. J. Am. Chem. Soc..

[61] Iwaoka M., Isozumi N. (2012). Hypervalent nonbonded interactions of a divalent sulfur atom. Implic. Protein Archit. Funct..

[62] Foley J.W., Song X., Demidova T.N., Jilal F., Hamblin M.R. (2006). Synthesis ad properties of benzo [a] phenoxazinium chalcogen analogues as novel broad-spectrum antimicrobial photosensitizers. J. Med. Chem..

[63] Andreesen J.R., Wagner M., Sonntag D., Kohlstock M., Harms C., Gursinsky T., Jäge J., Parther T., Kabisch U., Gräntzdöffer A. (1999). Various functions of selenols and thiols in anaerobic Gran-positive, amino acids-utilizing bacteria. Biofactors.

[64] Murray J.S., Lane P., Politzer P. (2009). Expansion of the sigma-hole concept. J. Mol. Model..

[65] Bujnicki J.M., Oudjama Y., Roovers M., Owczarek S., Caillet J., Droogmans L. (2004). Identification of a bifunctional enzyme MnmC involved in the biosynthesis of a hypermodified uridine in the wobble position of tRNA. RNA.

[66] Unrine J.M., Jackson B.P., Hopkins W.A. (2007). Selenomethionine biotransformation and incorporation into proteins along a simulated terrestrial food chain. Environ. Sci. Technol..

[67] Boyington J.C., Gladyshev V.N., Khangulov S.V., Stadtman T.C., Sun P.D. (1997). Crystal structure of formate dehydrogenase H: Catalysis involving Mo, molybdopterin, selenocysteine, and an Fe_4_S_4_ cluster. Science.

[68] Wu J., Gao W.X., Huang X.B., Zhou Y.B., Liu M.C., Wu H.Y. (2020). Selective [3+2] cycloaddition of cyclopropenone derivatives and elemental chalcogens. Org. Lett..

[69] Jiang J., Sheng J., Carrasco N., Huang Z. (2007). Selenium derivatization of nucleic acids for crystallography. Nucleic Acids Res..

[70] Kim J.Y., Carlson B.A., Xu X.M., Zeng Y., Chen S., Gladyshev V.N., Lee B.J., Hatfield D.L. (2011). Inhibition of selenocystein tRNA [Ser] Sec aminoacylation provides evidence that aminoacylation is required for regulatory methylation of this tRNA. Biochem. Biophys. Res. Commun..

[71] Salon J., Jiang J., Sheng J., Gerlits O.O., Huang Z. (2008). Derivatization of DNAs with selenium at 6-position of guanine for function and crystal structure studies. Nucleic Acids Res..

[72] Mishra K.K., Singh S.K., Kumar S., Singh G., Sarkar B., Madhusudhan M.S., Das A. (2019). Water-mediated selenium hydrogen-bonding in protein: PDB analysis and gas-phase spectroscopy of model complexes. J. Phys. Chem. A.

[73] Kulik K., Sadowska K., Wielgus E., Pacholczyk-Sienicka B., Sochacka E., Nawrot B. (2022). 2-Selenouridine, a Modified Nucleoside of Bacterial tRNAs, Its Reactivity in the Presence of Oxidizing and Reducing Reagents. Int. J. Mol. Sci..

[74] Takahashi K., Avissar N., Whitin J., Cohen H. (1987). Purification and characterization of human plasma glutathione peroxidase: A selenoglycoprotein distinct from the known cellular enzyme. Arch. Biochem. Biophys..

[75] Kim K., Jo C., Easwaramoorthi S., Sung J., Kim D.H., Churchill D.G. (2010). Crytallographic, Photophysical, NMR Spectroscopic and Reactivity Manifestations of the 8-Heteroaryl Effect in 4,4-Difluoro-8-(C4H3 X)-4-bora-3 a, 4 a-diaza-s-indacene (X = 0, S, Se)(BODIPY) Systems. Inorg. Chem..

[76] Howard D.L., Kjaergaard H.G. (2008). Hydrogen bonding to divalent sulfur. Phys. Chem. Chem. Phys..

[77] Kurihara K., Umezawa K., Donnelly A.E., Sperling B., Liao G., Hecht M.H., Arai R. (2023). Crystal structure and activity of a de novo enzyme, ferric enterobactin esterase Syn-F4. Proc. Natl. Acad. Sci. USA.

[78] Konidaris K., Daolio A., Pizzi A., Scilabra P., Terraneo G., Quici S., Murray J.S., Politzer P., Resnati G. (2022). Thiazolium salts as chalcogen bond donors. Cryst. Growth Des..

[79] Oliveira V., Kraka E. (2017). Systematic coupled cluster study of noncovalent interactions involving halogens, chalcogens, and pnicogens. J. Phys. Chem. A.

[80] Leonard K.A., Nelen M.I., Anderson L.T., Gibson S.L., Hilf R., Detty M.R. (1999). 2, 4, 6-Triarylchalcogenopyrylium dyes related in structure to the antitumor agent AA1 as in vitro sensitizers for the photodynamic therapy of cancer. J. Med. Chem..

[81] Jeong L.S., Tosh D.K., Choi W.J. (2009). Development of next generation 4’-selenonucleosides. Nucleic Acids Symp. Ser. Oxf. Univ. Press.

[82] Lancelot G. (1977). Hydrogen bonding between nucleic acid bases and carboxylic acids. J. Am. Chem. Soc..

[83] Kambampati R., Lauhon C.T. (2003). MnmA and IscS are required for in vitro 2-thiouridine biosynthesis in *Escherichia coli*. Biochemistry.

[84] Zeng L., Zhang T., Liu R., Tian W., Wu K., Zhu J., Wang Z., He C., Feng J., Guo X. (2023). Chalcogen-bridged coordination polymer for the photocatalytic activation of aryl halides. Nature.

[85] Zhao L., Zhao G., Du M., Zhao Z., Xiao L., Hu X. (2008). Effect of selenium on increasing free radical scavenging activities of polysaccharide extracts from a Se-enriched mushroom species of the genus *Ganoderma*. Eur. Food Res. Technol..

[86] Flohe L., Gunzler W.A., Schock H.H. (1973). Glutathione peroxidase: A selenoenzyme. FEBS Lett..

[87] Panzella L., Verotta L., Goya L., Ramos S., Martín M.A., Bravo L., Napolitano A., d’Ischia M. (2013). Synthesis and bioactivity profile of 5-S-lipoylhydroxytryrosol-based multidefense antioxidants with a sizeable (poly) sulfide chain. J. Agric. Food Chem..

[88] Rice L.M., Earnest T., Brunger A.T. (2000). Single-wavelength anomalous diffraction phasing revisited. Acta Crystallogr. Sect. D Biol. Crystallogr..

[89] Sierant M., Leszczynska G., Sadowska K., Komar P., Radzikowska-Cieciura E., Sochacka E., Nawrot B. (2018). *Escherichia coli* tRNA 2-selenouridine synthase (SelU) converts S2U-RNA to Se2U-RNA via S-geranylated-intermediate. FEBS Lett..

[90] Kaur M., Rob A., Caton-Williams J., Huang Z. (2013). Biochemistry of nucleic acids functionalized with sulfur, selenium, and tellurium: Roles of the single-atom substitution. Biochalcogen Chem..

[91] Berry M.J., Banu L., Larsen P.R. (1991). Type I iodothyronine deiodinase is a selenocysteine-containing enzyme. Nature.

[92] Strub M.P., Hoh F., Sanchez J.F., Strub J.M., Böck A., Aumelas A., Dumas C. (2003). Selenomethionine and selenocysteine double labeling strategy for crystallographic phasing. Structure.

[93] Roovers M., Oudjama Y., Kaminska K.H., Purta E., Caillet J., Droogmans L., Bujnicki J.M. (2008). Sequence–structure–function analysis of the bifunctional enzyme MnmC that catalyses the last two steps in the biosynthesis of hypermodified nucleoside mnm5s2U in tRNA. Proteins: Struct. Funct. Bioinform..

[94] Pfeiffer M., Bingemann R., Klein A. (1998). Fusion of two subunits does not impair the function of a [NiFeSe]-hydrogenase in the archaeon *Methanococcus voltae*. Eur. J. Biochem..

[95] Wagner M., Sonntag D., Grimm R., Pich A., Eckerskorn C., Söhling B., Andreesen J.R. (1999). Substrate-specific selenoprotein B of glycine reductase from *Eubacterium acidaminophilum*: Biochemical and molecular analysis. Eur. J. Biochem..

[96] Wuttig M., Schön C.F., Kim D., Golub P., Gatti C., Raty J.Y., Kooi B.J., Pendás Á.M., Arora R., Waghmare U. (2024). Metavalent or Hypervalent Bonding: Is There a Chance for Reconciliation?. Adv. Sci..

[97] Habib M., Kar M., Pal S., Sarkar P. (2019). Role of chalcogens in the exciton relaxation dynamics of chalcogenol-functionalized CdSe QD: A time-domain atomistic simulation. Chem. Mater..

[98] Detty M.R., Merkel P.B., Hilf R., Gibson S.L., Powers S.K. (1990). Chalcogenapyrylium dyes as photochemotherapeutic agens. 2. Tumor uptake, mitochondrial targeting, and singlet-oxygen-induced inhibition of cytochrome c oxidase. J. Med. Chem..

[99] Iwaoka M., Takemoto S., Okada M., Tomoda S. (2001). Statistical characterization of nonbonded S-O interactions in proteins. Chem. Lett..

[100] Höbartner C., Micura R. (2004). Chemical synthesis of selenium-modified oligoribonucleotides and their enzymatic ligation leading to an U6 SnRNA stem−loop segment. J. Am. Chem. Soc..

[101] Moustafa M.E., El-Saadani M.A., Kandeel K.M., Mansur D.B., Lee B.J., Hatfield D.L., Diamond A.M. (1998). Overproduction of selenocysteine tRNA in Chinese hamster ovary cells following transfection of the mouse tRNA [Ser] Sec gene. RNA.

[102] Moreno S., Fickl M., Bauer I., Brunner M., Rázková A., Rieder D., Delazer I., Micura R., Lusser A. (2022). 6-Thioguanosine monophosphate prodrugs display enhanced performance against thiopurine-resistant leukemia and breast cancer cells. J. Med. Chem..

[103] Rizwan M., Ali S., Rehman M.Z.U., Rinklebe J., Tsang D.C.W., Tack F.M.G., Abbasi G.H., Hussain A., Igalavithana A.D., Lee B.C. (2021). Effects of Selenium on the Uptake of Toxic Trace Elements by Crop Plants: A Review. Crit. Rev. Environ. Sci. Technol..

[104] Shigi N., Horitani M., Miyauchi K., Suzuki T., Kuroki M. (2020). An ancient type of MnmA protein is an iron–sulfur cluster-dependent sulfurtransferase for tRNA anticodons. RNA.

[105] Carrasco N., Ginsburg D., Du Q., Huang Z. (2001). Synthesis of selenium-derivatized nucleosides and oligonucleotides for X-ray crystallography. Nucleosides Nucleotides Nucleic Acids.

[106] Carrasco N., Buzin Y., Tyson E., Halpert E., Huang Z. (2004). Selenium derivatization and crystallization of DNA and RNA oligonucleotides for X-ray crystallography using multiple anomalous dispersion. Nucleic Acids Res..

[107] Korotkikh N.I., Rayenko G.F., Shvaika O.P., Pekhtereva T.M., Cowley A.H., Jones J.N., Macdonald C.L. (2003). Synthesis of 1,2,4-triazol-5-ylidenes and their interaction with acetonitrile and chalcogens. J. Org. Chem..

[108] Norberg J., Nilsson L. (1998). Solvent influence on base stacking. Biophys. J..

[109] Kikuchi N., Satoh K., Kurosawa R., Yaoita N., Elias-Al-Mamun M., Siddique M.A.H., Omura J., Satoh T., Nogi M., Sunamura S. (2018). Selenoprotein P promotes the development of pulmonary arterial hypertension: Possible therapeutic target. Circulation.

[110] Carugo O., Resnati G., Metrangolo P. (2021). Chalcogen bonds involving selenium in protein structures. ACS Chem. Biol..

[111] Begines P., Oliete A., Lopez O., Maya I., Plata G.B., Padron J.M., Fernandez-Bolanos J.G. (2018). Chalcogen-containing phenolics as antiproliferative agens. Future Medininal Chem..

[112] Scilabra P., Terraneo G., Resnati G. (2019). The chalcogen bond in crystalline solids: A world parallel to halogen bond. Acc. Chem. Res..

[113] Haruehanroengra P., Vangaveti S., Ranganathan S.V., Mao S., Su M.D., Chen A.A., Sheng J. (2020). Terpene Chain Length Affects the Base Pairing Discrimination of S-geranyl-2-thiouridine in RNA Duplex. Iscience.

[114] Haruehanroengra P., Zheng Y.Y., Ma G., Lan T.H., Hassan A.E., Zhou Y., Sheng J. (2022). Probing the Substrate Requirements of the In Vitro Geranylation Activity of Selenouridine Synthase (SelU). ChemBioChem.

[115] Durant P.C., Bajji A.C., Sundaram M., Kumar R.K., Davis D.R. (2005). Structural effects of hypermodified nucleosides in the *Escherichia coli* and human tRNALys anticodon loop: The effect of nucleosides s2U, mcm5U, mcm5s2U, t6A, and ms2t6A. Biochemistry.

[116] Bommisetti P., Young A., Bandarian V. (2022). Elucidation of the substrate of tRNA-modifying enzymes MnmEG leads to in vitro reconstitution of an evolutionarily conserved uridine hypermodification. J. Biol. Chem..

[117] Du Q., Carrasco N., Teplova M., Wilds C.J., Egli M., Huang Z. (2002). Internal derivatization of oligonucleotides with selenium for X-ray crystallography using MAD. J. Am. Chem. Soc..

[118] Mundlapati V.R., Sahoo D.K., Ghosh S., Purame U.K., Pandey S., Acharya R., Pal N., Tiwari P., Biswal H.S. (2017). Spectroscopic evidences for strong hydrogen bonds with selenomethionine in proteins. J. Phys. Chem. Lett..

[119] Arguello-Garcia R., Medina-Campos O.N., Perez-Hernandez N., Pedraza-Chaverri J., Ortega-Pierres G. (2010). Hypochlorous acid scavening and activities of thioallyl compounds from garlic. J. Agric. Food Chem..

[120] Rosenfield R.E., Parthasarathy R., Dunitz J.D. (1977). Directional preferences of nonbonded atomic contacts with divalent sulfur. 1. Electrophiles and nucleophiles. J. Am. Chem. Soc..

[121] Reid R.C., Yau M.K., Singh R., Lim J., Fairlie D.P. (2014). Stereoelectronic effects dictate molecular conformation and biological function of heterocyclic amides. J. Am. Chem. Soc..

[122] Fick R.J., Kroner G.M., Nepal B., Magnani R., Horowitz S., Houtz R.L., Scheiner S., Trievel R.C. (2016). Sulfur-oxygen chalcogen bonding mediates adomet recognition in the lysine methyltransferase SET7/9. ACS Chem. Biol..

[123] Roman M., Jitaru P., Barbante C. (2014). Selenium biochemistry and its role for human health. Metallomics.

[124] Wang R., Vangaveti S., Ranganathan S.V., Basanta-Sanchez M., Haruehanroengra P., Chen A., Sheng J. (2016). Synthesis, base pairing and structural studies of geranylated RNA. Nucleic Acids Res..

[125] Masuda R., Kimura R., Karasaki T., Sase S., Goto K. (2021). Modeling the catalytic cycle of glutathione peroxidase by nuclear magnetic resonance spectroscopic analysis of selenocysteine selenenic acids. J. Am. Chem. Soc..

[126] Yang S.J., Hwang S.Y., Choi H.Y., Yoo H.J., Seo J.A., Kim S.G., Kim N.H., Baik S.H., Choi D.S., Choi K.M. (2011). Serum selenoprotein P levels in patients with type 2 diabetes and prediabetes: Implications for insulin resistance, inflammation, and atherosclerosis. J. Clin. Endocrinol. Metab..

[127] Tsutsumi R., Saito Y. (2020). Selenoprotein P; P for plasma, prognosis, prophylaxis, and more. Biol. Pharm. Bull..

[128] Benz S., López-Andarias J., Mareda J., Sakai N., Matile S. (2017). Catalysis with chalcogen bonds. Angew. Chem. Int. Ed..

[129] Lee S.R., Kim J.R., Kwon K.S., Yoon H.W., Levine R.L., Ginsburg A., Rhee S.G. (1999). Molecular cloning and characterization of a mitochondrial selenocysteine-containing thioredoxin reductase from rat liver. J. Biol. Chem..

[130] Boggon T.J., Shapiro L. (2000). Screening for phasing atoms in protein crystallography. Structure.

[131] Sheng J. (2009). Synthesis, Structure and Function Studies of Selenium and Tellurium Derivatized Nucleic Acids. Ph.D. Thesis.

[132] Watabe S., Makino Y., Ogawa K., Hiroi T., Yamamoto Y., Takahashi S.Y. (1999). Mitochondrial thioredoxin reductase in bovine adrenal cortex: Its purification, properties, nucleotide/amino acid sequences, and identification of selenocysteine. Eur. J. Biochem..

[133] Nahar S., Singh A., Morihiro K., Moai Y., Kodama T., Obika S., Maiti S. (2016). Systematic evaluation of biophysical and functional characteristics of selenomethylene-locked nucleic acid-mediated inhibition of miR-21. Biochemistry.

[134] Stadtman T. (1983). New biologic functions—Selenium-dependent nucleic acids and proteins. Fundam. Appl. Toxicol..

[135] Tamura T., Stadtman T.C. (1996). A new selenoprotein from human lung adenocarcinoma cells: Purification, properties, and thioredoxin reductase activity. Proc. Natl. Acad. Sci. USA.

[136] Sasamori T., Sasaki T., Takeda N., Tokitoh N. (2005). Reactions of a Germacyclopropabenzene with Elemental Chalcogens: Synthesis and Structures of a Series of Stable 2 H-Benzo [c][1,2] chalcogenagermetes. Organometallics.

[137] Nauser T., Steinmann D., Grassi G., Koppenol W.H. (2014). Why selenocysteine replaces cysteine in thioredoxin reductase: A radical hypothesis. Biochemistry.

[138] Numata T., Ikeuchi Y., Fukai S., Adachi H., Matsumura H., Takano K., Murakami S., Inoue T., Mori Y., Sasaki T. (2006). Crystallization and preliminary X-ray analysis of the tRNA thiolation enzyme MnmA from *Escherichia coli* complexed with tRNAGlu. Acta Crystallogr. Sect. F Struct. Biol. Cryst. Commun..

[139] Uemura K., Nobori H., Sato A., Toba S., Kusakabe S., Sasaki M., Tabata K., Matsuno K., Maeda N., Ito S. (2023). 2-Thiouridine is a broad-spectrum antiviral nucleoside analogue against positive-strand RNA viruses. Proc. Natl. Acad. Sci. USA.

[140] Olieric V., Rieder U., Lang K., Serganov A., Schulze-Briese C., Micura R., Dumas P., Ennifar E. (2009). A fast selenium derivatization strategy for crystallization and phasing of RNA structures. RNA.

[141] Vishal A., Adhav B.P., Saikrishnan K. (2022). Probing the Directionality of S O/N Chalcogen Bond and Its Interplay with Weak C-H O/N/S Hydrogen Bond Using Molecular Electrostatic Potential. J. Phys. Chem. B.

[142] Waters M.L. (2004). Aromatic interactions in peptides: Impact on structure and function. Pept. Sci. Orig. Res. Biomol..

[143] Hendrickson W.A., Horton J.R., LeMaster D.M. (1990). Selenomethionyl proteins produced for analysis by multiwavelength anomalous diffraction (MAD): A vehicle for direct determination of three-dimensional structure. EMBO J..

[144] Zhang W., Szostak J.W., Huang Z. (2016). Nucleic acid crystallization and X-ray crystallography facilitated by single selenium atom. Front. Chem. Sci. Eng..

[145] Wang W., Walter M.J., Brodholt J.P., Huang S., Petaev M.I. (2023). Chacogen isotopes reveal limited volatile contribution from late veneer to Earth. Sci. Adv..

[146] Schneider T.F., Werz D.B. (2010). Caged Chalcogens: Theoretical Studies on a Tetracoordinated Oxonium Dication and Its Higher Homologues. Org. Lett..

[147] Guo X., An X., Li Q. (2015). Se-N Chalcogen Bond and Se-X Halogen Bond Involving F2C=Se: Influence of Hybridization, Substitution, and Cooperativity. J. Phys. Chem. A.

[148] Yu Y., Liu Z., Luo L.Y., Fu P.N., Wang Q., Li H.F. (2019). Selenium uptake and biotransformation in *Brassica rapa* supplied with selenite and selenate: A hydroponic work with HPLC speciation and RNA-sequencing. J. Agric. Food Chem..

[149] Li Y., Meng L., Sun C., Zeng Y. (2020). Organocatalysis by halogen, chalcogen, and pnictogen bond donors in halide abstraction reactions: An alternative to hydrogen bond-based catalysis. J. Phys. Chem. A.

[150] Kyogoku Y., Lord R.C., Rich A. (1966). Hydrogen bonding specificity of nucleic acid purines and pyrimidines in solution. Science.

[151] Mita Y., Nakayama K., Inari S., Nishito Y., Yoshioka Y., Sakai N., Sotani K., Nagamura T., Kuzuhara Y., Inagaki K. (2017). Selenoprotein P-neutralizing antibodies improve insulin secretion and glucose sensitivity in type 2 diabetes mouse models. Nat. Commun..

[152] Buzin Y., Carrasco N., Huang Z. (2004). Synthesis of selenium-derivatized cytidine and oligonucleotides for X-ray crystallography using MAD. Org. Lett..

